# Traumatic perforated diverticulum of the fourth duodenal portion: First case report and literature review

**DOI:** 10.1016/j.amsu.2020.07.026

**Published:** 2020-07-21

**Authors:** Valentina Palumbo, Maria Sofia, Edoardo Mattone, Rosario Bonaccorso, Tommaso Guastella, Gaetano La Greca, Domenico Russello, Saverio Latteri

**Affiliations:** a Department of Medical and Surgical Sciences and Advanced Technologies “G.F. Ingrassia”, University of Catania, Via S. Sofia 84, 95123, Catania, Italy; b General Surgery, Cannizzaro Hospital, via Messina 829, 95126, Catania, Italy

**Keywords:** Duodenal diverticulum, Perforation, Abdominal trauma, Case report

## Abstract

**Introduction:**

Duodenal rupture following blunt abdominal trauma is rare, and traumatic rupture of duodenal diverticula is exceptional. However, duodenum is the second most frequent location of intestinal diverticula following colon. Duodenal diverticula are common but only in few cases they are symptomatic due to the onset of complications such as inflammation, hemorrhage, or perforation. Perforation, although rare, especially post-trauma, is the most serious life threatening complication.

**Case presentation:**

We report the case of a patient who, 24 hours after a blunt trauma secondary to a car accident, complained symptoms related to the perforation of a diverticulum of the fourth portion of the duodenum. A computed tomography was performed and extraluminal fluid-air collection was identified. During emergent laparotomy, a fourth portion perforated duodenal diverticulum was diagnosed, and resected. The recovery was uneventful.

**Discussion:**

Diagnosis of perforated duodenal diverticulum represents a challenge in diagnosis and few guidelines exist about the management of this rare occurrence, especially in a traumatic setting. The present case is the first report of traumatic perforated diverticulum of the fourth duodenal portion.

**Conclusion:**

Surgery still remain the most common approach in the treatment of this pathology, including diverticulectomy and primary repair.

## Highlights

• Duodenal diverticula are a frequent, but they are usually asymptomatic until they develop complications, as perforation.
• Traumatic perforation of duodenum is very infrequent and the traumatic rupture of duodenal diverticula is extremely rare.
• Perforation, usually involves the second part of the duodenum.
• The present case is the first report of traumatic perforated diverticulum of the fourth duodenal portion.
• Surgery remain the most common approach in the treatment of complicated duodenal diverticula.

## 1 Introduction

Duodenum is the second most frequent location, after colon, for diverticula in the digestive tract. The incidence rate at autopsy is 22% and an increase in prevalence is seen with age [1,2]. The most part (60%) of duodenal diverticula are located in the second portion of the duodenum, followed by third portion (30%) and few (8%) are present in the fourth segment of duodenum [[3], [4], [5], [6]]. Patients with duodenal diverticula are usually asymptomatic, and the diagnosis is frequently made during endoscopic retrograde cholangiopancreatography (ERCP) [2,3]. Only the 5% of patients with duodenal diverticula can present symptoms related to complications, such as hemorrhage, obstruction, compression of biliopancreatic structures, inflammation and perforation [3,7]. Perforation is an uncommon complication, usually as an evolution of diverticulitis, it can be rarely caused by iatrogenic causes, foreign bodies, enterolithiasis and finally by trauma [8]. Traumatic perforation of duodenum is very infrequent and the traumatic rupture of duodenal diverticula is extremely rare, and it represent a diagnostic challenge and difficult surgical problem. The authors present the first case of a traumatic perforation of the duodenal fourth portion diverticulum managed successfully by surgery. This work has been reported in line with the SCARE criteria [9].

## 2 Case Presentation

**Fig. 1:**
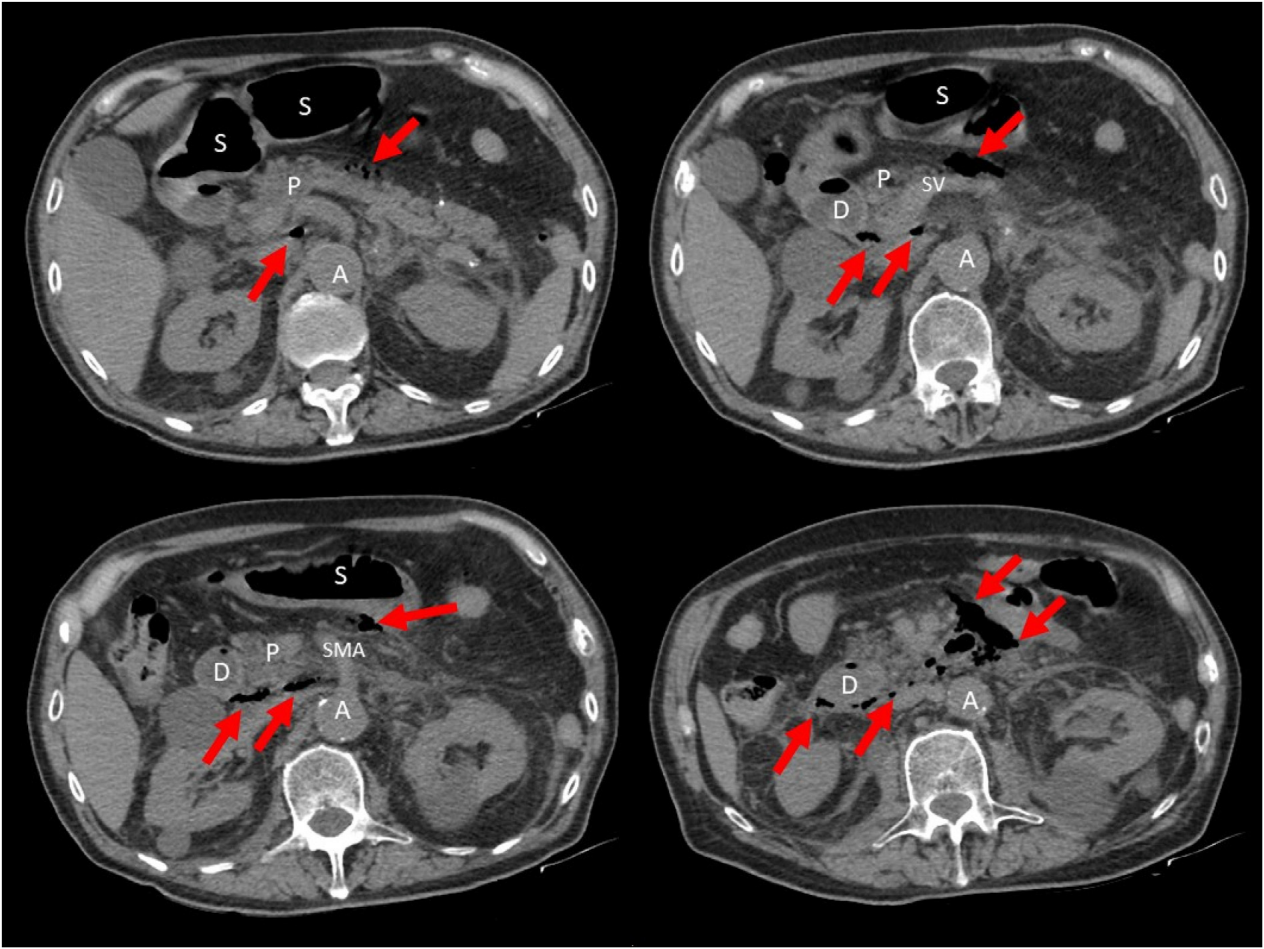
Computed tomography of the abdomen showing retroperitoneal fluid with free air (red arrows), suggestive of perforation. (P: pancreas; A: aorta; S: stomach; D: duodenum; SV: splenic vein; SMA: superior mesenteric artery). (For interpretation of the references to colour in this figure legend, the reader is referred to the Web version of this article.)

**Fig. 2:**
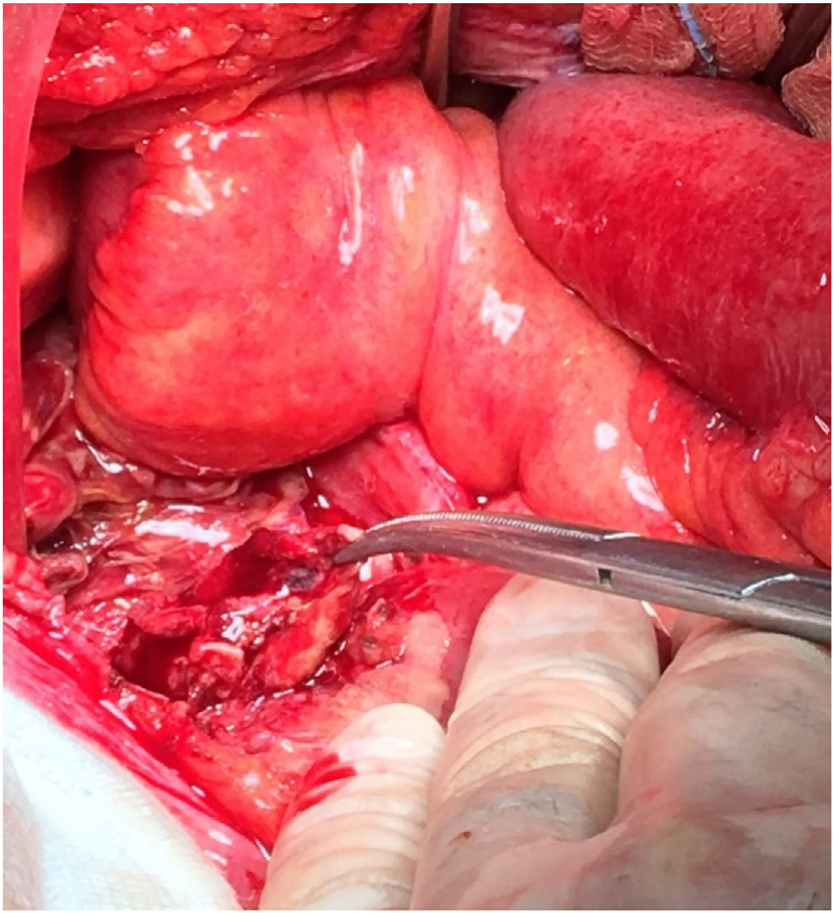
The instrument indicates the little ischemic area close to the first jejunum.

A 82-year-old man was admitted to the Emergency Department following a blunt trauma secondary to low energy car accident. He reported head trauma and sprain trauma of cervical spine. He was asymptomatic, but he remained in the Emergency Department to complete the clinical observation after head trauma. After 10 hours, the patient complained abdominal pain and vomiting. His vital signs were temperature of 36.8 °C, heart rate of 78/min and blood pressure of 130/70 mmHg. Physical examination revealed tender in the epigastric and middle area with signs of diffuse peritoneal irritation. Blood tests showed: white blood cells 11,900/mm3, hemoglobin 13.4 g/dL, platelets 219,000/mm3. The differential count of white blood cells was neutrophil 86.1%, lynphocytes 8% and monocytes 5.6%. C-reactive protein was 1.56 mg/dL. Liver function tests were normal with a slight increase of aspartate-alanine transaminase (78 U/l), whereas lactate dehydrogenase was high (702 U/l). Renal function test and amylase concentrations were normal. Abdominal X-ray showed no intraperitoneal or subdiaphragmatic free air. Computed tomography (CT) scan of abdomen showed a fluid and air filled collection in the retroperitoneal area near duodenum supporting the diagnosis of traumatic duodenal perforation (Fig. 1). An emergency laparotomy was performed. After the mobilization of the duodenum by Kocher maneuver, inspection of duodenum was carried on its third portion, without signs of pathology. An ischemic area was observed through a small laceration of peritoneum close to the first jejunal loop (Fig. 2). Incision of this peritoneum revealed a leakage of enteric fluid and air, but its origins was not already clear. Finally, after a fine dissection, the perforated diverticulum was detected in the posterior wall of the fourth portion of the duodenum. Diverticulectomy was performed by linear cutting stapler. Hydropneumatic test was carried out to detect some leakages. There was no evidence of injuries to other abdominal organs. Nasogastric tube and two abdominal drains were placed. The postoperative course was uneventful and the patient was discharged without complications. Histological analysis supported the initial diagnosis of a perforated pseudo-diverticulum of the duodenum.

## 3 Discussion

In 1710 Pierre Chomel was the first to describe duodenal diverticula [10]. They can be congenital or acquired, these are the most common including 90% of duodenal diverticula. Congenital diverticula present all layers of duodenal wall. While acquired diverticula or pseudodiverticula represent a herniation of the mucosa and submucosa through a weakness in the muscular wall of the duodenum, where blood vessels penetrate or near the papilla. This explains why the area near of papillae in the second duodenum is the most common site for this pathology [7]. Duodenal diverticula are asymptomatic until they develop complications, which are: inflammation, hemorrhage, compression of neighboring organs, pancreatitis and at list perforation [2]. Perforation is the rarest complication, with only about 200 cases reported in the literature [11], with potentially dramatic sequelae and high mortality (8%–34%) [12]. Diverticulitis is the most common cause of perforation, followed by enterolithiasis, iatrogenic perforation due to ERCP, ulceration and trauma [4,5,13].

Traumatic duodenal rupture, with or without diverticula, is very infrequent, representing 4% of abdominal traumatic lesions [14]. The rupture following blunt trauma is usually related to motor vehicle accidents or accidental fall [15,16], less frequent during sport [17]. A literature review reveals 14 cases, including the present, of traumatic perforation of duodenal diverticula [13,16,[18], [19], [20], [21], [22], [23], [24], [25], [26], [27], [28]] (Table 1). All cases of traumatic perforation of duodenal diverticulum, reported in literature, were located in the second part of the duodenum [16,28]. The present case is the first report of traumatic perforated diverticulum of the fourth duodenal portion. The mechanism of intestinal rupture is still unclear. Three mechanical models can explain the rupture [29]. First, a force on the anterior abdominal wall that compress the duodenum against the spine (e.g. the steering wheel against the epigastrium during car accident). Second, an acceleration or deceleration motion, causing a stretching on the fixed intestinal portion, can create tears near the points of attachment (e.g. fall from great heights). Third, a duodenal explosion due to high intraluminal pressures produced when the pylorus and the fourth duodenal part are suddenly closed at the same time. In our case, the compression of the diverticulum against lumbar vertebra during deceleration has caused a sudden increased in intraluminal pressure, which resulted in the outbreak of the diverticulum. Duodenal traumas are often associated to other abdominal organ injuries, but exceptionally traumatic perforation of duodenal diverticulum is always isolated [16,30]. Only in the case reported by Angus et al., the perforation of duodenal diverticulum is associated with other organ injury as renal fracture and splenic laceration [27]. The moderated severity of the blunt trauma, as reported in the other descripted cases, and the weakness point in the diverticulum can explain, because the traumatic rupture of duodenal diverticulum is always an isolated pathology.

**Table 1:** Reported cases of traumatic perforation of duodenal diverticulum.

Author	Y	Sex	Age	Cause of trauma	Part of Duodenum	Treatment	drainage	Morbidity/Mortality
Braband (18)	1960	F	54	Fall	2nd	Handsewn diverticulectomy	Yes	Duodenal fistula
Graudins (19)	1970	F	70	Fall	2nd	Handsewn diverticulectomy	Yes	Pancreatitis, renal failure Death
Souza Junior Ade (20)	1996	M	49	Road accident	2nd	Handsewn diverticulectomy	Yes	Duodenal fistula Death
Poostizadeh (21)	1997	F	72	Road accident	2nd	Cholangiography, diverticuloraphy, gastrostomy, feeding jejunostomy	Yes	Death
Atmani (22)	2002	F	83	Road accident	2nd	Cholangiography, diverticulectomy, lateral duodenostomy	Yes	None
Valenzuela Martinez (23)	2006	F	73	Fall fromheight	2nd	Handsewn diverticulectomy	Yes	None
Fowler (24)	2008	F	45	Fall frombucket	2nd	Stapled diverticulectomy, pyloric exclusion andgastrojejunostomy	Yes	None
Nazim (25)	2009	F	84	Road accident	2nd	Stapled diverticulectomy, decompressive gastrostomy and jejunostomy, feeding jejunostomy	Yes	Pneumonia
Metcalfe (13)	2010	M	58	Fall fromheight	2nd	Handsewn diverticulectomy with omental patch.	Yes	Septicemia, wound infection, hemorrhage
Wedemeyer (26)	2012	F	79	Impact against barrier	2nd	Stapled diverticulectomy, duodenotomy	Yes	None
Angus (27)	2013	F	64	Road accident	2nd	Stapled diverticulectomy, decompressive gastrostomy	Yes	Pulmonary embolism
Majerus (16)	2015	F	65	Road accident	2nd	Stapled diverticulectomy	Yes	Pulmonary atelectasis, Retroperitoneal collection
Albin (28)	2015	F	65	Road accident	2nd	diverticulectomy	Yes	None
Present Case	2020	M	82	Road accident	4th	Stapled diverticulectomy	Yes	None

Perforated duodenal diverticulum represents a diagnostic challenge. Patients usually complain acute abdominal pain without evidence of peritoneal signs due to a retroperitoneal perforation [2,8]. Other vague and non-specific symptoms such as fever, nausea and vomiting are similar to those associated to perforated peptic ulcer, acute cholecystitis and pancreatitis. Blood samples are also unspecific. A history of abdominal blunt trauma can bring a suspicion of perforation [16]. Symptoms usually arise early in traumatic perforation, but they can be delayed until several days after the trauma. In the most part of the patients, conventional radiological examinations show no abnormalities [31,32], and abdominal CT scan is often requested without any suspicion for complicated duodenal pathology. CT scan can be considered the most useful diagnostic tool in duodenal diverticulum perforation, as it can shows the signs of perforation: air bubbles, extraluminal fluid, retroperitoneal abscess, thickened bowel wall, mesenteric fat stranding [31]. In our case, a history of trauma and the sudden abdominal pain suggested a delayed traumatic organ lesion. The CT scan revealed retroperitoneal free air and fluid collection hinting a duodenal perforation, but it did not identified the diverticulum. Only in 13% of the cases, a correct preoperative diagnosis could be made [7], often, only at laparotomy, an accurate diagnosis can be established [12].

Surgery is the standard treatment for perforated duodenal diverticulum, even if there are some reports of conservative treatment, called "Taylor's approach" for upper gastrointestinal perforation [12,29]. This approach is reserved for patients in a good clinical condition without signs of sepsis, and it includes bowel rest, antibiotic therapy, nasogastric suction, parenteral nutrition, and sometimes percutaneous or endoscopic drainage can be combined to the treatment [8,29]. Of course, if patient deteriorates, surgery become mandatory [12]. Due to the infrequence of perforated duodenal diverticulum, surgical treatment guidelines are lacking. The most common procedures includes diverticulectomy with transverse duodenal closure, manually or mechanically in single or double layer [12,16,33]. We performed a diverticulectomy with linear cutting stapler on a duodenal wall, raising an optimal result, as there was no inflammation and edema on the surrounding tissue. In all reported case of traumatic duodenal perforation, a drainage next to the duodenal closure was left in order to detect any early anastomotic leak [16,28]. In relation on the place of diverticula, the presence of biliary tract obstruction or the severity of retroperitoneal inflammation, other more complex procedures may be needed [32,33]. These include duodenal diversion, pyloric exclusion, gastroenteric anastomois, Petzer tube duodenostomy, segmental duodenal resection or even a Whipple's procedure [32,33]. In recent years, this pathology has been treated with success also by laparoscopic procedures [34].

The complications after surgery include duodenal fistula or leak, intraabdominal abscess, sepsis, iatrogenic injury to the biliary tract, and acute pancreatitis [7]. The onset of these complications can lead up to 31% of mortality rate [3]. From literature reported case, three on thirteen patients died.

## 4 Conclusions

The perforation of a diverticulum of the fourth duodenal portion is an extremely rare event, especially when it happens after a trauma. There are no clear guidelines that could help the surgeon who has to deal with this surgical emergency. Overall, early surgery is the treatment of choice, because if a traumatic perforation is not diagnosed and treated early, it could be life threatening for the patient. Our experience supports this and suggests that the surgical treatment is effective and needful to obtain good results in the outcome of patients affected from traumatic perforation of duodenal diverticulum.

## Ethical approval

NA. This is a case report study.

## Funding

This research did not receive any specific grant from funding agencies in the public, commercial, or not-for-profit sectors.

## Guarantor

All authors have read and approved the manuscript and accept full responsibility for the work.

## Registration of research studies

NA. This is a case report study.

## Provenance and peer review

Not commissioned, externally peer review.

## Informed consent

Informed written consent has been obtained and all identifying information is omitted.

## Declaration of competing interestCOI

Authors have no conflict of interest to disclose.

## Author contributions

MS, VP - drafting of abstract, drafting of manuscript.

VP, EM, SL - acquisition of data, analysis and interpretation of data.

TG, RB, GLG - patient management and care.

DR, GLG, SL, MS - critical revision of the manuscript.
